# Data on minerals and crystallinity index of quartz in rock samples collected from Paleolithic archaeological site of Attirampakkam, Tamil Nadu

**DOI:** 10.1016/j.dib.2021.107571

**Published:** 2021-11-13

**Authors:** A. Tamilarasi, V. Sathish, S. Manigandan, A. Chandrasekaran

**Affiliations:** Sri Sivasubramaniya Nadar College of Engineering (Autonomous), Chennai, Tamilnadu 603110, India

**Keywords:** Paleolithic archaeological rock, FT-IR, Crystallinity index, XRD

## Abstract

In the present work, rock samples were collected from Paleolithic archaeological site of Attirampakkam, Tamil Nadu, India to assess the mineralogical composition using Fourier transform infrared-spectroscopic (FT-IR) technique and X-Ray Diffraction Spectrometry (XRD). The quartz, kaolinite, montmorillonite, calcite, orthoclase, microcline and illite minerals are identified in rock samples and crystallinity index of quartz (SiO_2_) is estimated for all the samples by comparing the ratio of intensity of the characteristic peak at 778 and 695 cm^–1^ using FT-IR spectrum. In rock samples, calculated crystallinity index of quartz is greater than the 1 from FT-IR spectrum and it shows that the distribution is disordered in nature. Additionally, some more minerals such as hematite and rutile are identified in rock samples by X-ray diffraction technique. This extensive study shows that archeological rock samples are wide variation in mineral composition.

**Specifications Table**

**Table:** 

Subject	Earth and Planetary Sciences
Specific subject area	Minerals in earth materials
Types of data	Table, graph, figures
How data were acquired	FT-IR, XRD
Data format	Raw analyzed
Parameters for data collection	Minerals analysis, crystallinity index for Quartz
Description of data collection	The mineralogical characterization was carried out using the FT-IR and XRD techniques. The crystallinity index was calculated by using the formula: $\text{Crystalline}\;\text{index}\;\text{of}\;\text{quartz}=\;\frac{I_{778}}{I_{695}}$ Where, I_778_, I_695_ is the intensity of the absorption band and suffix is representing the frequency of the band.
Data source location	Region: Attirampakkam archaeological site, Tamilnadu, Country: India Latitude and Longitude: 13° 13′ 55.59″ N and 79° 52′ 48.36″ E Samples: Rocks
Data accessibility	Data is with this article.

## Value of the Data

• Data could be used as a baseline for analyzing the minerals.
• Data used to determine the difference between archeological rock and other rocks.
• This data provides the information on the nature of quartz in archeological rock samples.

## 1 Data Description

**Fig. 1:**
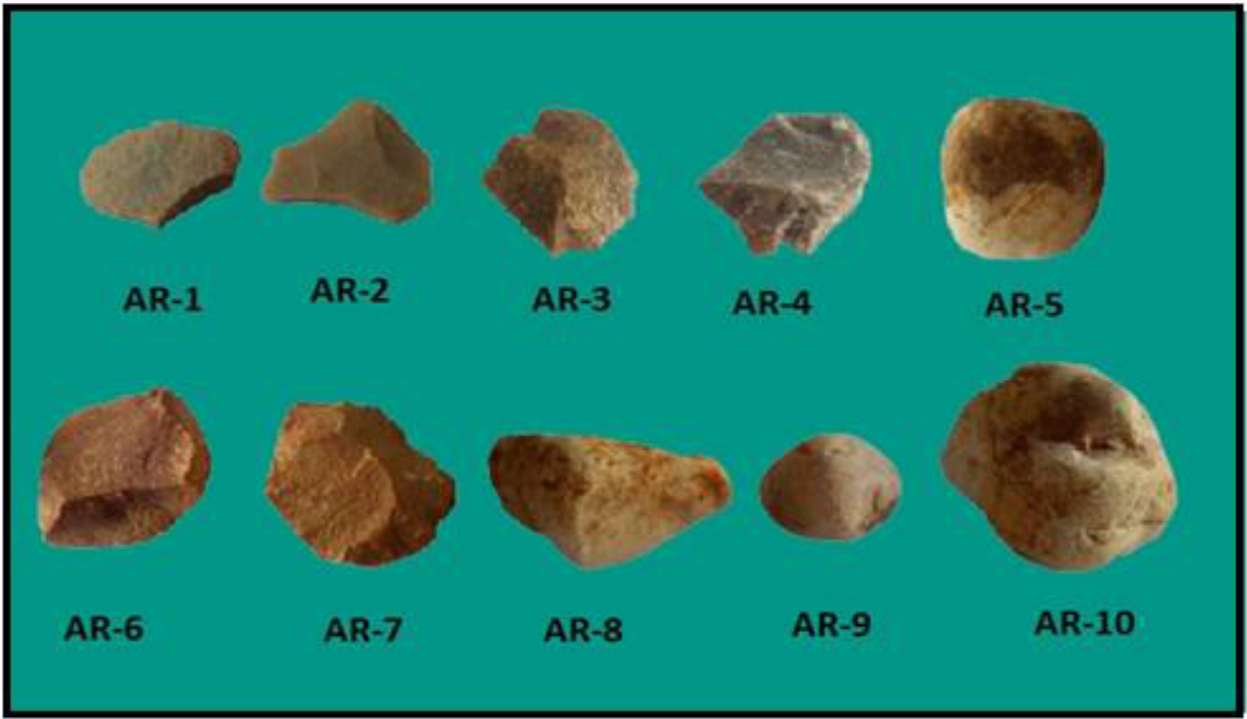
The collected rock samples of Archelogical site of Attirampakkam, Tamil Nadu, India.

**Fig. 2:**
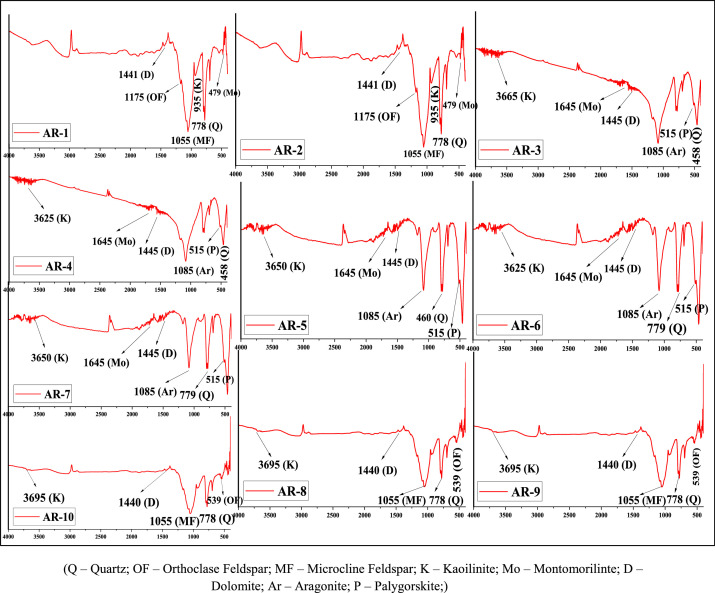
FT-IR spectrum [X-axis – Wavenumber (cm^−1^); Y-axis Transmittance (%)] with observed frequencies and identified minerals in rock samples of Archelogical site of Attirampakkam, Tamil Nadu, India.

**Fig. 3:**
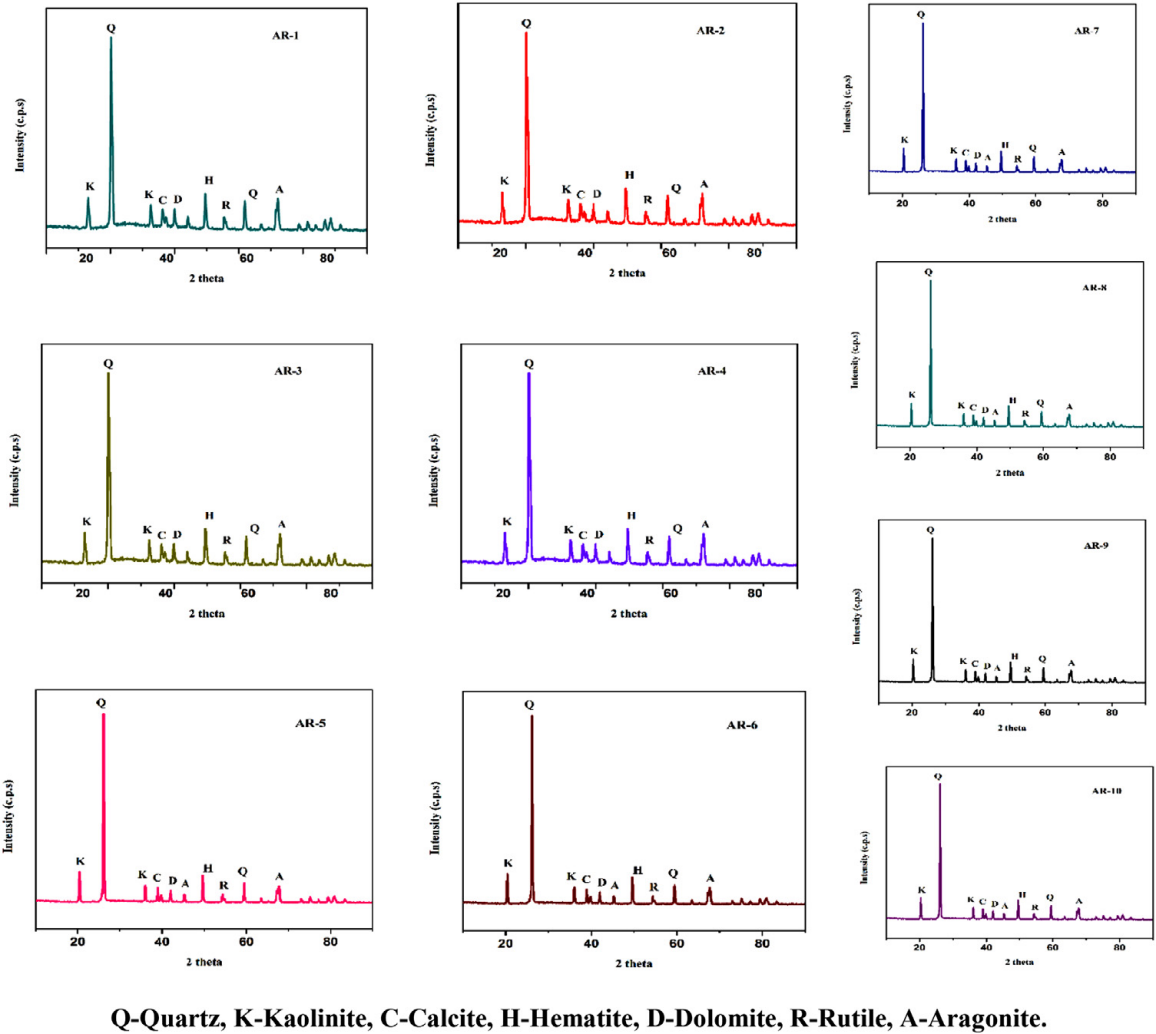
XRD spectrum of rock samples of Archelogical site of Attirampakkam, Tamil Nadu, India.

The rock samples were collected from the Paleolithic archaeological site of Attirampakkam, Tamilnadu, India and shown in Fig. 1. Minerals are basic constituent of rocks which makes the baseline for earth materials. Rocks are formed mainly due to dissolution of minerals. Rock formation affects if mineral phase are not significant Hence, the minerals are important and its assessment is essential to understand the baseline of rocks. The mineralogical characterizations of the samples were measured using both the FT-IR and XRD techniques. FT-IR and XRD spectrums are recorded and given in Figs. 2 and 3, respectively. The absorbed frequency of the infra spectra of the rock samples are tabulated and the corresponding minerals of these absorbed peaks are identified and given Table 1. And also the crystallinity index of quartz (SiO_2_) was estimated for all the samples by comparing the ratio of intensity of the characteristic peak at 778 and 695 cm^–1^ with the corresponding ratio for a standard sample are shown in Table 2. Also, the list of peak intensity for quartz is given Table 3.

**Table 1:** Observed absorption frequency in the region of 400–4000 cm^−1^.

	Silicate mineral	Feldspar		Clay Mineral		Carbonate Minerals		
Sample ID	Quartz	Orthoclase	Microcline	Kaoilinite	Montomorilinte	Dolomite	Aragonite	Palygorskite
AR1	695, 778, 1615, 1870	1175	1055	535,935, 3625	479	1441		-
AR2	695, 778, 1615, 1870	1175	1055	535,935, 3625	479	1441		-
AR3	458, 695, 779, 1164, 1620	-	-	3625, 3665	1645	1445	1085	515
AR4	458, 695, 779, 1085,1164, 1620	-	-	3625, 3665	1645	1445	1085	515
AR5	460,695,779, 1875	-	-	3625,3650,	3440, 1645	1445	1085, 1480	515
AR6	460,695,779, 1875	-	-	3625, 3650	3440, 1645	1445	1085, 1480	515
AR7	460,695,779, 1875	-	-	3625,3650,	3440, 1645	1445	1085, 1480	515
AR8	695, 778, 1165	435,539	1055	940, 3695	-	1440	-	-
AR9	695, 778, 1165	435,539	1055	940, 3695	-	1440	-	-
AR10	695, 778, 1165	435,539	1055	940, 3695	-	1440	-

**Table 2:** Crystallinity index of quartz for the peak 778 cm^−1^.

Sample ID	Crystallinity index
AR1	1.1518
AR2	1.0900
AR3	1.1407
AR4	1.0929
AR5	1.0535
AR6	1.2459
AR7	1.1759
AR8	1.2140
AR9	1.1727
AR10	1.2424

**Table 3:** List the peak intensity for quartz in the rock samples.

Sample I D	2 theta (2θ)	d-spacing
AR1	26.14, 60.03	3.414, 1.534
AR2	26.14	3.414
AR3	26.14, 60.03	3.414, 1.534
AR4	26.14	3.414
AR5	26.14, 60.03	3.414, 1.534
AR6	26.14	3.414
AR7	26.14, 60.03	3.414, 1.534
AR8	26.14	3.414
AR9	26.14, 60.03	3.414, 1.534
AR10	26.14	3.414

## 2 Experimental Design, Materials and Methods

### 2.1 Sample collection and preparation of FT-IR

The ten rock samples with different shape and texture are collected in the sampling site and after cleaning the samples are grounded then it will packed for the further investigations. These grounded samples are sieved using 63µm for all the particles are having same size for accurate result [1]. In FT-IR analysis, KBr compressed-pellet method was used to identify the minerals in these rock samples and mineral confirmation and additional minerals are identification was carried out by XRD [2].

### 2.2 Crystallinity index

The crystallinity index of quartz is estimated quartz is major mineral present in the rock samples. On the other side, crystallinity of quartz will give a clear indication on the crystalline forms of other minerals because quartz is the mineral, which crystallizes last [3]. The crystallinity index is calculated using the formula [4].

$$\text{Crystalline}\;\text{index}\;\text{of}\;\text{quartz}=\;\frac{I_{778}}{I_{695}}$$

Where, I_778_ is the intensity of absorption band around 778 cm^−1^ due to the vibrations in tetrahedral site symmetry and I_695_ is the intensity of the absorption band around 695cm^−1^ due to the vibrations in octahedral site symmetry [5].

## Declaration of Competing Interest

The author declares that they have no known competing financial interests.

## Appendix Supplementary materials

Image, application 1

Image, application 2

## References

[1] Mullainathan S., Nithiyanantham S. (2016). FTIR spectroscopic studies of rock sediments in Namakkal, Tamil Nadu, South India, for vegetations. Environ. Earth Sci..

[2] Ravisankar R., Eswaran P., Rajalakshmi A., Chandrasekaran A., Dhinakaran B. (2012). Beach rocks from the south east coast of Tamilnadu, India: a spectroscopic study. Adv. Appl. Sci..

[3] Ramasamy V., Suresh G. (2009). Mineral characterization and crystalline nature of quartz in Ponnaiyar River sediments, Tamilnadu, India. Am. Eurasian J. Sci. Res..

[4] Cannane N.O.A., Rajendran M., Selvaraju R. (2013). FT-IR spectral studies on polluted soils from industrial area at Karaikal, Puducherry State, South India. Spectrochim. Acta Part A Mol. Biomol. Spectrosc..

[5] White J.L., Roth C.B., Klute A. (1996). Methods of Soil Analysis Part I–Physical and Mineralogical Methods.

